# Supplementary figures and images for: Automated morphological phenotyping using learned shape descriptors and functional maps: A novel approach to geometric morphometrics
Source: PLoS Comput Biol. 2023 Jan 19;19(1):e1009061. doi: 10.1371/journal.pcbi.1009061 (PMC9970057; doi:10.1371/journal.pcbi.1009061)

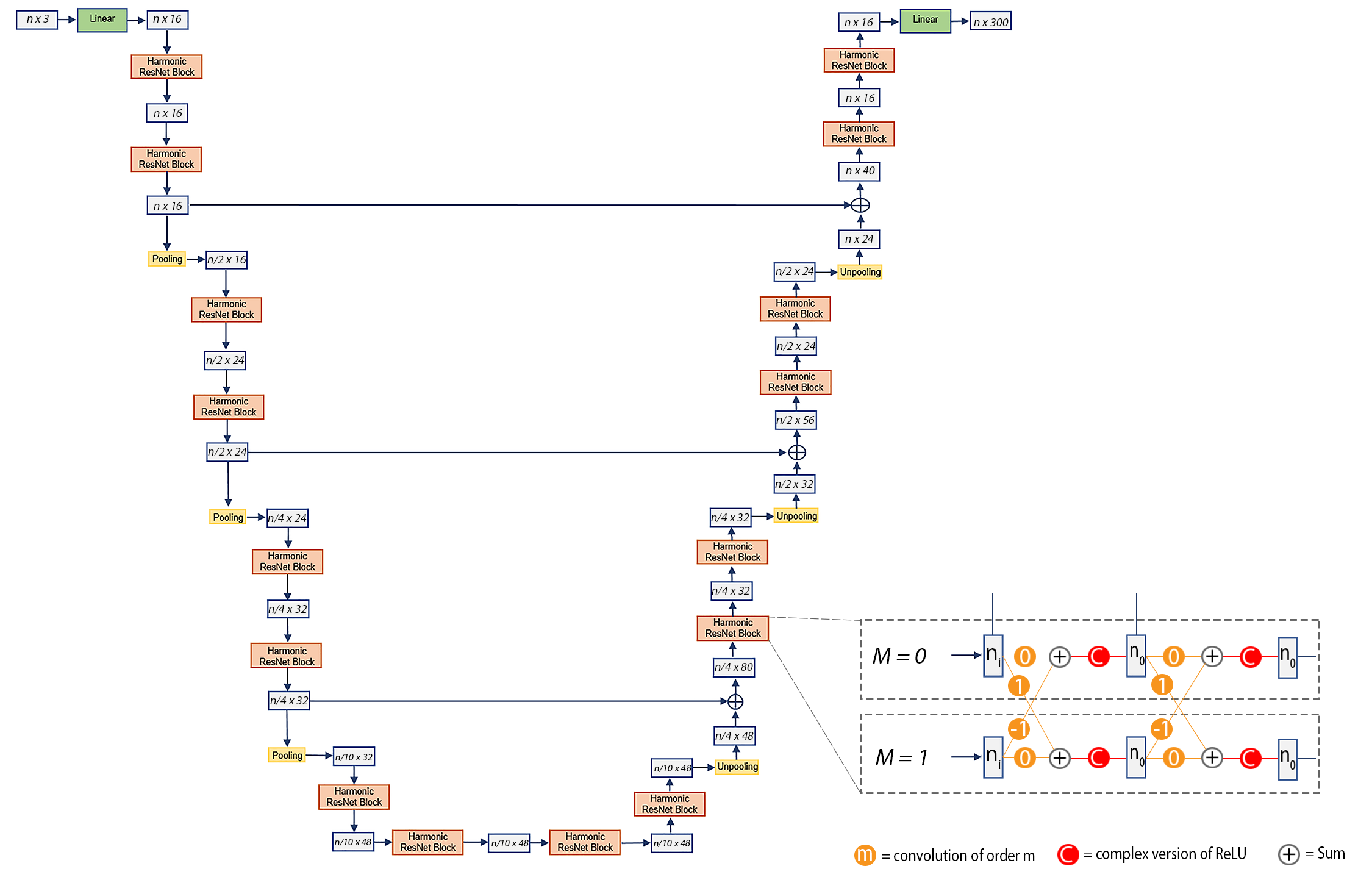

Supplement: S1 Fig — Adapted from Wiersma et. al., 2020. On the left is the U-ResNet structure with three pooling levels, on the right, is a detailed description of the ResNet block. (TIF) [file pcbi.1009061.s001.tif]

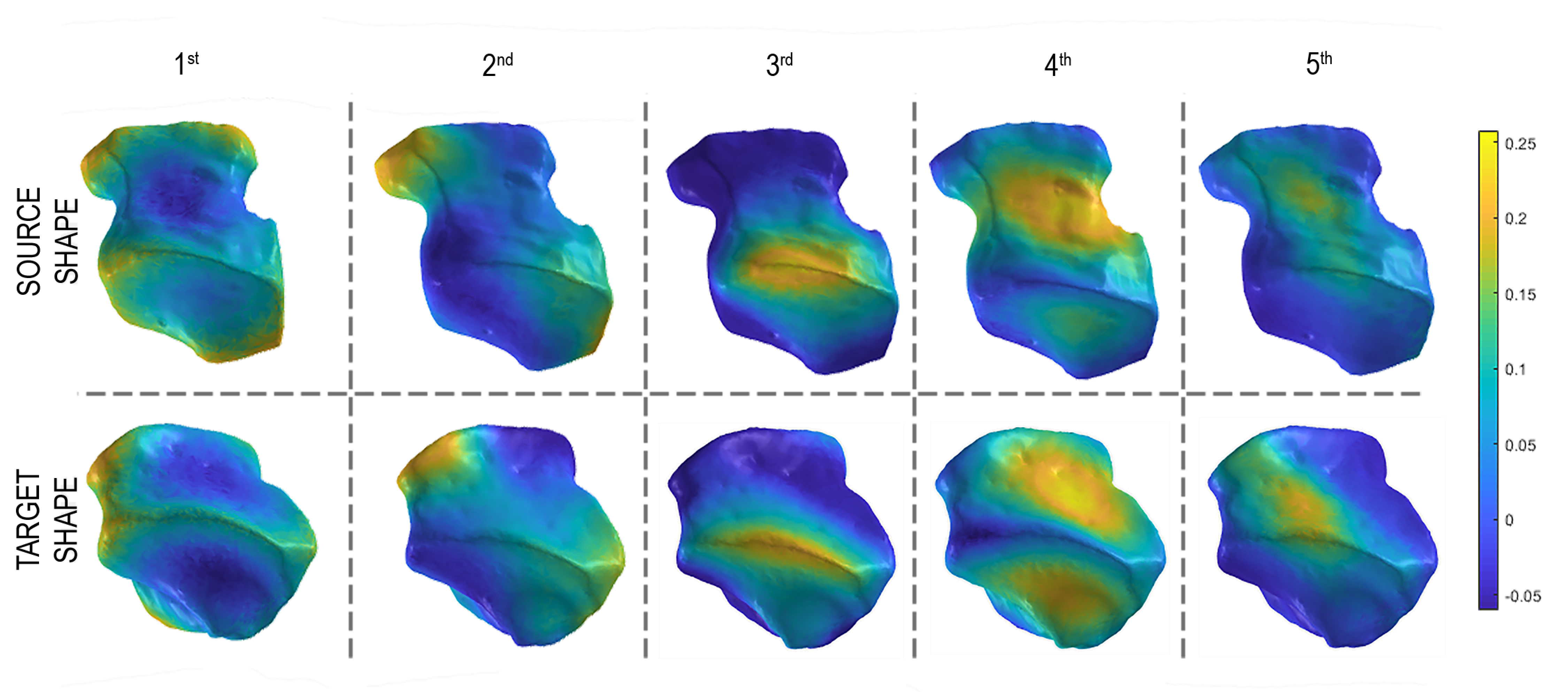

Supplement: S2 Fig — The first five dimensions of the spectral descriptor learned with our HSN feature extractor. Corresponding/homologous regions on Hylobates source (above) and Pan target (below) shapes are similar in color. These learned descriptors yield high-quality FM correspondences. (TIF) [file pcbi.1009061.s002.tif]

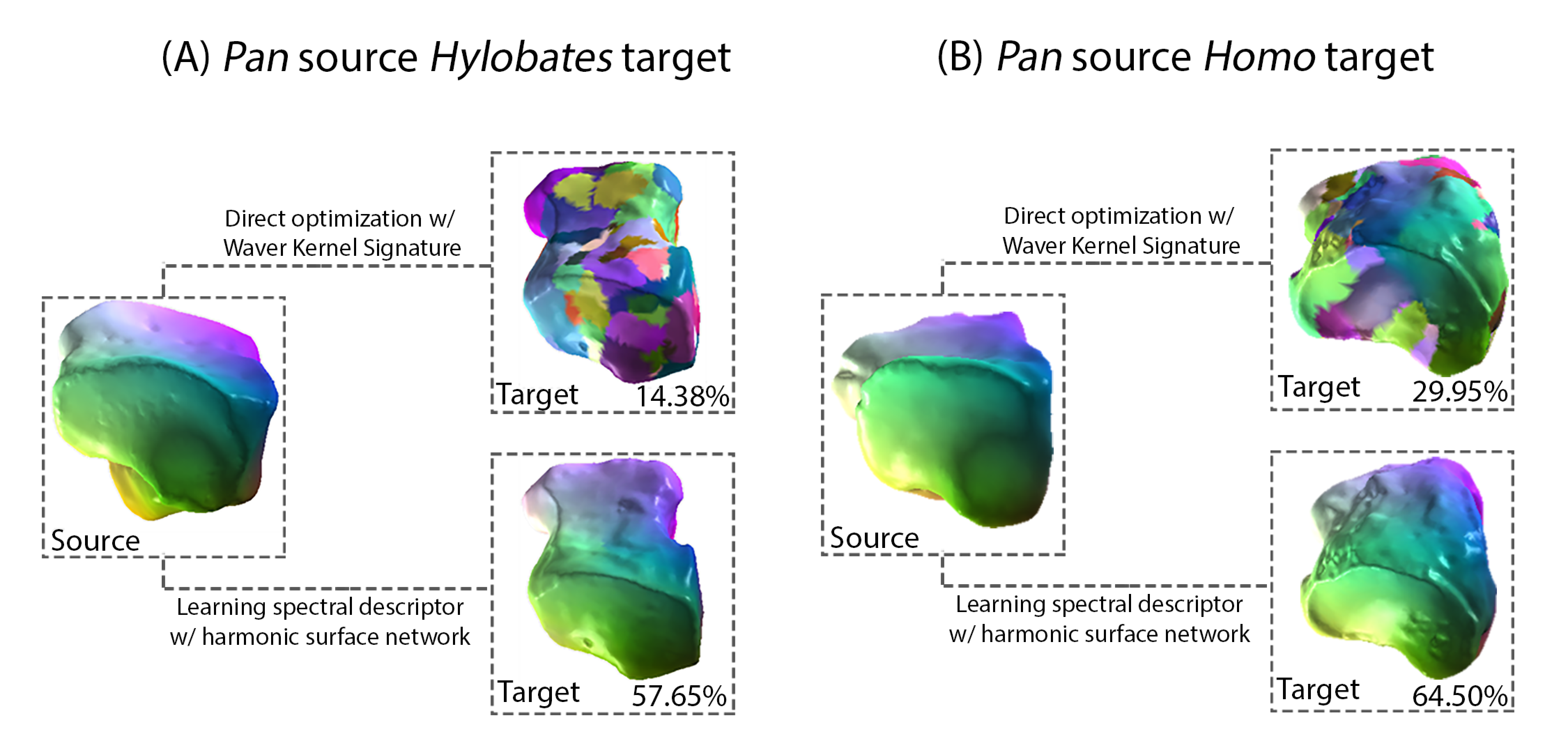

Supplement: S3 Fig — Point-to-point maps between source Pan shape and Hylobates target shape (left to right) obtained via direct optimization using WKS (above) and our learned spectral descriptor (below) [25]. Source and target shapes are remeshed to 12,000 vertices in both experiments. WKS were computed using 200 Laplace-Beltrami eigenfunctions. (TIF) [file pcbi.1009061.s003.tif]

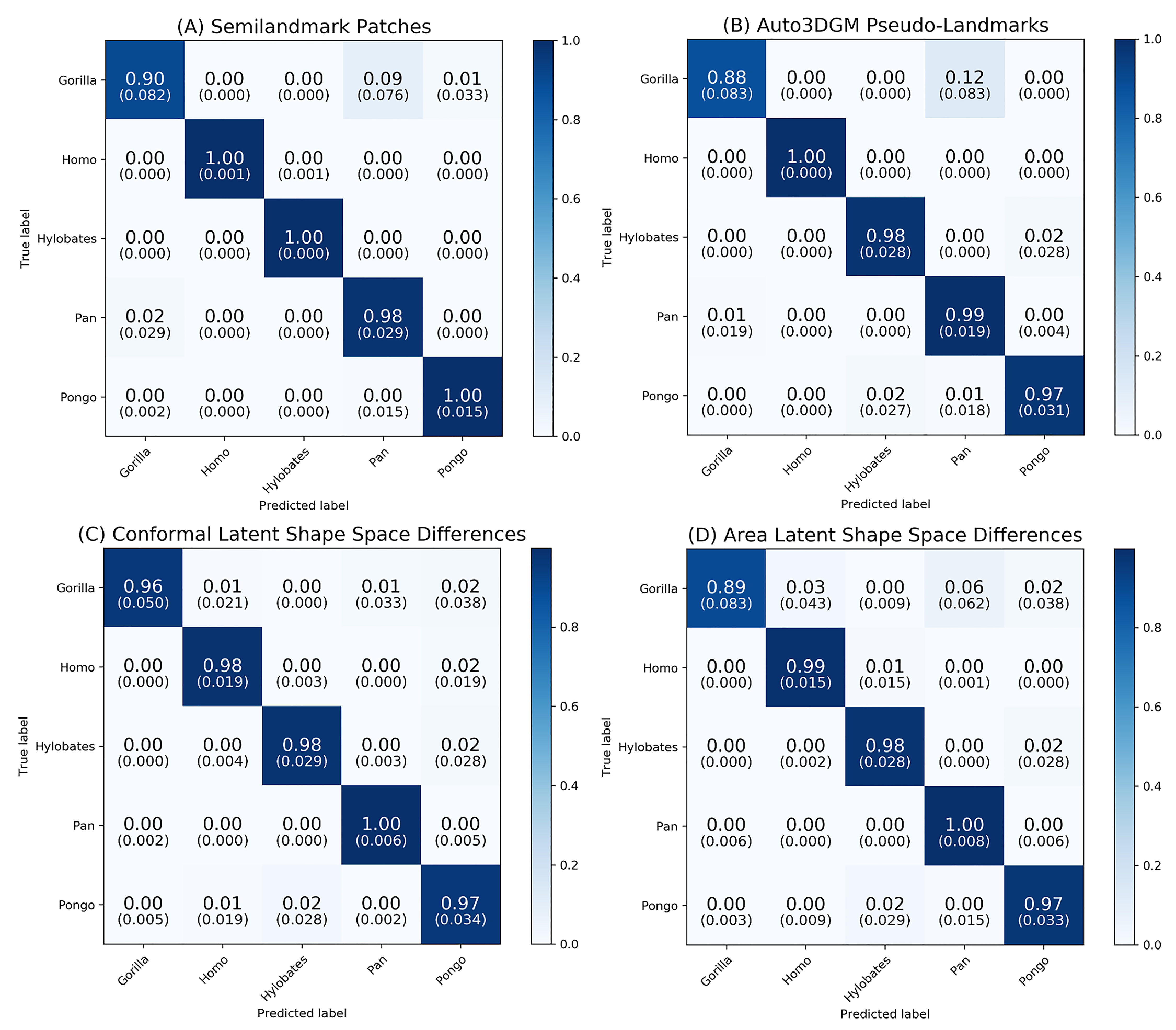

Supplement: S4 Fig — Confusion matrices of multinomial logistic regression prediction for each representation of hominoid cuboid shape. In (A) and (B) use principal components of Procrustes-aligned semilandmark patches and pseudolandmarks generated by auto3DGM as independent variables, respectively. Independent variables in (C) and (D) are principal components of conformal and area-based LSSDs, respectively. PCs account for 95% shape variance for each representation, and stratified K-Fold cross-validation provides accuracies with standard deviations in parentheses. (TIF) [file pcbi.1009061.s004.tif]
