# Supplementary material for: Automated morphological phenotyping using learned shape descriptors and functional maps: A novel approach to geometric morphometrics
Source: PLoS Comput Biol. 2023 Jan 19;19(1):e1009061. doi: 10.1371/journal.pcbi.1009061 (PMC9970057; doi:10.1371/journal.pcbi.1009061)
Supplement: Attachment — Submitted filename: Response to Reviewers.pdf [file pcbi.1009061.s007.pdf]

ILLINOIS

COLLEGE OF LIBERAL ARTS & SCIENCES
Department of Anthropology
109 Davenport Hall, MC-148
607 S. Matthews Ave.
Urbana, IL 61801-3636

Dear Editors Xu and Ma,

On behalf of all of the authors, we thank you and the reviewers for your thorough review of our manuscript. We have carefully considered and addressed the issues suggested by the reviewers and have detailed our responses below. As requested we have attached both a marked-up version of the original manuscript and a clean version incorporating all changes.

We trust that our changes and commentary in the responses below have adequately addressed all of the reviewers' concerns.

Sincerely,

Oshane O. Thomas
Ph.D. Candidate | Department of Anthropology
University of Illinois at Urbana-Champaign

Revisions

Reviewer One

This is an ambitious paper that describes a new shape analysis approach that tries to establish functional correspondences between whole triangular meshes (as opposed to landmark configurations, as traditionally done in morphometrics) using descriptor learning. While I find the rationale behind the approach sound, I cannot say that I found this approach successful. My comments to the authors will concentrate on three main issues: generalizability, accuracy, and robustness.

1. Thank you for your recognition of our ambitious goals. Your thorough review helps us to respond to make our paper stronger for journal readers. We are grateful for your efforts.

First, starting with the simplest issue: generalizability. The approach in question claims to be a general approach for shape analysis that is aimed at replacing traditional morphometric tools. Yet, the entire manuscript is based on a single dataset, composed of a single morphological structure (102 hominoid cuboids). The structure in question, the cuboid, is also topologically very simple and asymmetric, which greatly facilitates the estimation of shape correspondences. Overall, I find this lacking. At the very least, an effort needs to be made to demonstrate that the approach is indeed general. There is a wealth of triangular meshes freely available online through databases such as Morphosource, Facebase, etc. Skulls, teeth, long bones, etc, represent just a few of the multiple skeletal structures that are abundant in such databases.

   1.1. To address the reviewer's concern we have applied the method to investigating shape variation in mouse humeri and in primate medial cuneiforms. These datasets differ from the cuboid dataset in overall size, resolution, and topological complexity. The use of these two additional datasets demonstrates that our method is generalizable to a range of different applications. We describe these datasets in section 4.1 (Data acquisition and preprocessing) and introduce results in a new section 2.4 (Robustness to topological differences and mesh quality) that are then discussed briefly in paragraph 7 of the discussion.

Secondly, I think it is worth talking about accuracy. The manuscript makes two attempts to measure accuracy in the predictions. On one side, it looks at species classification accuracy, which I find misleading and indirect (as will be explored in my next point).

1.2. We agree that accuracy is a fundamental issue. We explore both of the described types of accuracy because each is useful in demonstrating different issues. Accurate species classification illustrates a successful application of the method to a common biological problem. Evaluating accuracy in comparing landmark-based methods to the functional map approach used here is not simple since functional maps assess morphological complexity using functions that describe the totality of the external shape, rather than an array of discrete landmarks and inferred semilandmarks. Nevertheless, we have implemented a new assessment of accuracy by finding mesh vertices that are closest to manually-placed landmarks, and we compare scaled linear distances obtained using morphVQ to those obtained using auto3DGM. Our results demonstrate a comparable or higher level of accuracy as evidenced by similar or slightly lower distances for morphVQ than auto3DGM. We have incorporated new figures and discussion sections to reflect these changes.

To be clear, we feel that this measure of accuracy is a simplistic approximation since it involves only an assessment of a small number of discrete points, and detracts from the more comprehensive quantification of surface morphology enabled by morphVQ which is not attainable using other landmark-based methods.

In the field of representation learning, considering the learned features (embeddings of the original data) and use such features for some downstream tasks (e.g. regressions, classifications) is a popular and solid way of validating the power of the model that generates those features from the input data (cuboid in this case). Since one of the goals in this paper is to demonstrate that this proposed model is able to learn meaningful features (conformal/area-based differences) that represent the original data, which perfectly agrees with the point in representation learning. As a result, providing such classification tasks is not misleading. To make it clear, in the paper, we not only address that the model is capable of (p2p mapping), but also (conformal difference highlights.)

More importantly, the manuscript also compares manual to automated representations. In my view, this second measurement of accuracy is much more direct and important given the goals of this manuscript. When considering this second measurement of accuracy, I find the approach quite error-prone. As reported on the main text (Page 6), representations derived from the approach presented in this paper are moderately-to-poorly correlated

with manual representations (r =0.621). I find this quite low and, in my view, that would be enough to render the approach unusable for practical purposes.

1.3. We have addressed this concern in the discussion section. Our conformal and area-based differences are complementary decompositions that represent variation in whole shapes, not just landmarks or pseudolandmarks. For this reason, we do not expect them to be highly correlated with the point-based representations. While this correlation gives us some idea that these data are similarly structured, the new estimates of landmark positions mention in review response 1.2 are a better indication of this similarity of content since those same landmark positions that result in the distance matrix used for this correlation can be predicted well from our new representation.

While the manuscript attempts to benchmark this in terms of Auto3DGM (claiming it is the only other such approach in the literature), this is factually incorrect, as a large amount of automated approaches became available in recent years (some of which have readily available implementations). To cite a few:

[1] Devine, J., Aponte, J.D., Katz, D.C., Liu, W., Vercio, L.D.L., Forkert, N.D., Marcucio, R., Percival, C.J. and Hallgrímsson, B., 2020. A registration and Deep Learning approach to automated landmark detection for geometric morphometrics. Evolutionary Biology, 47(3), pp.246-259.
[2]Toussaint, N., Redhead, Y., Vidal-García, M., Lo Vercio, L., Liu, W., Fisher, E.M., Hallgrímsson, B., Tybulewicz, V.L., Schnabel, J.A. and Green, J.B., 2021. A landmark-free morphometrics pipeline for high-resolution phenotyping: application to a mouse model of Down syndrome. Development, 148(18), p.dev188631.
[3] Maga, A.M., Tustison, N.J. and Avants, B.B., 2017. A population level atlas of Mus musculus craniofacial skeleton and automated image‐based shape analysis. Journal of anatomy, 231(3), pp.433-443.
[4]Porto, A., Rolfe, S.M. and Maga, A.M., 2020. ALPACA: a fast and accurate approach for automated landmarking of three-dimensional biological structures. bioRxiv.
[5]Frank, Lawrence R., Timothy B. Rowe, Doug M. Boyer, Lawrence M. Witmer, and Vitaly L. Galinsky. "Unveiling the third dimension in morphometry with automated quantitative volumetric computations." (2021).

1.4. We incorporated Auto3DGM as this was the only method available during development and analysis of the data. The reviewer is correct that a few other methods have been presented more recently. However, in evaluating this list of methods many require a manually-digitized template and others implement learning methods that are supervised to achieve desired

classifications. Auto3DGM is the only comparative method that does not require supervision or a template and that is why we used it for comparison.

We have updated the manuscript with reference to these other papers and expanded the justification for our use of auto3DGM as the benchmark for comparison.

Finally, my third and last point is related to robustness. In my view, not enough was done to guarantee the robustness of the approach. In particular, the main task being explored in the manuscript is the task of species classification. The manuscript attempts to argue that this approach obtains high classification accuracy (higher than Auto3DGM). I find this misleading, as it is obvious from all the plots (and confusion matrices) that the classification task at hand was extremely "easy". By "easy", I mean that species differences are clearly pronounced and, consequently, all methods present very high accuracy levels (0.90 and above). In my view, this classification test tells us little about the robustness of the method.

1.5. It is not entirely clear to us what the reviewer means by 'robustness', if this is in reference to this method's ability to work well in difficult situations with potentially noisy data, we have demonstrated that with the use of new datasets. If robustness is in reference to our method's ability to pick up on small differences in shape between specimens, then the fact that we can predict landmark positions with as much accuracy as a well established method indicates that notable differences in shape are accounted for.

Classification is not aimed at showing the robustness of this model, it's used to demonstrate that our model learns biologically informative features.

Compared to 3DGM, since our approach achieves high accuracy, it means the model can generate features from the input data in a more biologically meaningful way.

Altogether, the aims here are laudable, but a number of important issues can be found with the approach. Until a robust test of the method is done, it will be difficult to get behind it.

1.6. We are grateful to this reviewer for their critiques and comments and believe that our responses have addressed their concerns and the resulting revisions have made the manuscript and method more compelling.

Reviewer Two

General Overview

This paper presents a pipeline system, the Morphological Variation Quantifier (morphoVQ) an analysis pipeline (or collection of processing steps) that is meant to automate the extraction of variables from 3D scans of objects, typically structures of biological objects, hominoid cuboid bones in this case.

The current common approach to extracting variables from these types of 3D scans is 3D landmark based geometric morphometrics (3DGM), which requires a skilled and knowledgeable human operator to digitize landmark and/or semi-landmark locations on each specimen, which is a costly process. There is also at least one currently existing automated approach to extracting variables for comparison, Auto3DGM.

The paper compares the performance of the newly developed analysis pipeline morphoVQ to the results obtained using the other two methods. While it is difficult in this situation to have a "ground truth", the human operator based 3DGM (with or without the use of semi-landmarks) at least represents something of a standard or accepted state of the art approach (albeit labor intensive).

The paper looks at the classification of specimens using K-means, logistic regression, a Naïve Bayes classifier and a Linear Discriminant Analysis on PCA axes scores derived from the 6 distinct data sets. Visual representations of where the variance is most pronounced are also presented.

Data, and Data Subsets

The data was 102 laser surface scans of hominoid cuboid bones, which had vertex counts from 2000-39,000 points, these were interpolated or reduced to a standardized 12,000 vertices using a common algorithm. One hopes that the possible impact of this standardization of vertex count has been studied previously, and/or looked at some detail with regards to it's impact on subsequent analyses.

2. The number of vertices in the mesh is an important factor in the analysis. The nature of the mesh is dependent on analytical method. For example if we were using wave-kernel signatures or SURFMNET with shot-descriptors would require more than double the number of vertices - and this would increase computational load. One feature of our method is the use of Harmonic Surface Networks (HSN), and we learn the descriptors we can use fewer vertices and therefore process a larger dataset. We considered three properties of the mesh network (i) ensuring adequate surface coverage, (ii) maintaining spectral properties and (iii) having a small enough mesh size to enable training.

   12000 vertices was empirically chosen because it was high-enough to enable quantification of morphology and low enough to ensure computational effectiveness. For

the mouse and medial cuneiform datasets included in this revision we were able to use even smaller meshes with 6000 vertices without loss of coverage or effects on spectral properties.

Both 3DGM and Auto3DGM were carried out on this data, producing two variations on each method. In 3DGM one approach bused 21 LMs, while the second added SLMs for a total of 130 points. AutoGM was set to create two sets, 256 and 512 pseudo-landmarks.

2.1. As described in the results the 256-points were used only for bone alignment in the morphVQ pipeline. Shape variables were not analyzed from the 256 points. The 512-point dataset was used to generate the shapes attributable to auto3DGM.

The morphoVQ method (see discussion below) was used to extract two sets, one conformal (angular based) and one area based LSSDs (LSSD- Latent Shape Space Difference). The differences in shapes are thus decomposed by the Functional map network (FMN) approach into measures that represent differences in areas of the surfaces and differences in relative orientation or angle. It appears that two morphoVQ data sets are used, one representing conformal changes and one representing area differences

From my perspectives the use of this decomposition of the shape differences in morphoVQ into the conformal (angular) and area-based result needs more discussion. The authors compare the 3DGM and Auto3D methods at different data point densities (numbers of points) to the morphoVQ data sets which differ not in the number of points used, but in the nature or decomposition of the shape differences.

In the 3DGM and Auto3D approaches, the two set sets in each case differ in the number of points, but in the morphoVQ, the sets used differ not in the number of points or variables used, but rather in the nature of shape differences (conformal or areal) employed. The sub-setting of the 3 data types is not consistent across the 3 analysis methods.

Why are the conformal and area results in morphoVQ split into two discrete data sets and then analyzed separately? They represent different types of shape differences, why are they treated separately? Surely it is possible to combine the area and conformal data about shape changes into a unified analysis when carrying out the classification task?

The ability to split shape differences into the different types of alteration (conformal and area) may be of some real use (as decompositions often are), but it is not clear why data set differing in decomposition (the morphoVQ) should be compared to data differing in point density (3DGM and Auto3D). Please explain in detail why this is done, and/or show comparisons among data sets that differ simply in point density.

If morphVQ cannot be used without the decomposition step, or if the two decomposition products cannot or should not be treated separately.

2.2. The morphVQ method generates two different representations, conformal (reflecting angular changes on surface) and area-based (quantifying relative-sizes of portions of the bone). These differences are described in the introduction. These two methods allow identification of where and why bone shapes differ, thus we present figures to illustrate the cuboid regions where conformal and area-based regions differ between genera. See figures 8 and 9.

This means of representing shape differences as resulting from differential distortions of area or angularity provides a new method to address a longstanding problem in comparative morphology.

Shape Space Comparison

The shape spaces are compared using Pearon's r.

What variables are used in the computation of Pearson's r? Is this a multivariate form of R, using one set of shape variables to predict another? Or is it the r based on estimated intra-specimen distances? I think this section needs to be a bit more explicit on exactly how this was done.

It was rather interesting that the range of r values reported was very narrow, ranging from 0.639 to 0.616. This narrow strikes me as a bit odd, in that all the variations in data produce such a narrow range of correlations around a moderate level of correlation. Is there any way to see what correlation arises from a randomization process on this data?

If all the correlations were uniformly high (up over 0.9), I would understand the compressed range, but the grouping of all the correlations around the same value seems odd. It does appear that the area and conformal results (split by the decomposition) are being compared to the non-decomposed 3DGM and Auto3D results, is that correct?

2.3. We use within-specimen distances to compute the Pearson's r values and have made this more clear in the methods and results.

The range of correlation values we observe is empirical. We do not claim that values are limited to this range. Performing randomization tests to explain the distribution of correlation values is beyond the scope of the paper and would distract from the main points that we are trying to make.

Yes the area and conformal results are compared to non-decomposed auto3DGM results. This is one reason why we believe these correlations are relatively high (in contrast to reviewer 1) who incorrectly views them as being somewhat low.

Classification Results

This looks to be well done, I'm glad to see a fold-based cross validation used here.

Again, I'd like to see an analysis of the combined Area and conformal data sets here, it might show even higher performance.

If you revise this section, I'd suggest trying a quick random forest classifier and/or an SVM classifier, they seem to outperform the earlier methods, at least with some data sets. Once you have the data reduced to PCA scores, running a couple of other classifier methods is a pretty trivial operation and may give you some higher rates.

2.4. While the methods suggested by this reviewer might be interesting. We applied methods that had previously been used in shape analyses, and we prefer the assumptions made by discriminant function analysis, Naive Bayes, and K-means. We see no a priori reason to include additional classification methods that would be unlikely to produce substantially different results.

We are grateful that this reviewer recognizes the value in the classification analysis as this is an important application to this type of data analysis. We have not supplemented with additional analyses at this time as this would inordinately increase the length of the manuscript.

Figures

In figures 6 and 7, I assume the columns in these figures have the same labels as they do in figure 5? Please add the column labels to the figures, it just makes them easier to interpret.

2.5. Thank you for this observation, the columns are in fact different between 5 the next two figures.

Figures 6 and 7 are showing Area-based and conformal based differences using the 3 different methods. How were the area and conformal information extracted from the 3DGM and Auto3D methods? Did I miss this in the ms? Please explain this in detail, or simply let me know if I missed this.

2.6. FIgures 6 and 7 illustrate distinctiveness functions for morphVQ that showing the regions of high shape variability between hominoid groups based on either conformal or area based metrics. We don't extract area-based and conformal information from auto3DGM and 3DGM data.

The morphoVQ method

The morphoVQ method is a pipeline of series of operations to extract Latent Shape Space Differences (LSSDs) through a lengthy set of operations. The Deep HSN UResNet Feature extractor process used as a feature extractor is using some non-linear elements, so this process is fairly non-linear.

The now-standard 3DGM also has a non-linear operation, the division by a centroid size measure. A lot of effort in the development of GM methods focused on the nature and impact of non-linear operations on the distribution of variance in these systems. When scaling by size, the consistent use of centroid size at least meant a consistent non-linear effect on the data, uniform across studies. That will not be the case with the morphoVQ method.

Early work on 3DGM was in the context of a statistical world where resampling/permutation and cross validation were just starting to see substantial use. Use of GM data with traditional statistical methods with substantial distributional assumptions embedded within them meant that non-linearity in data processing (such as division by centroid size or other scaling factors) produced alterations in the underlying distributions of variance that posed substantial concerns, particularly when using traditional statistical approaches with distributional assumptions.

I think some discussion of the impact of the extensively non-linear nature of this method is warranted, perhaps there is some available literature in this area that could be cited. This is particularly critical for applied workers. Figure 1 does help, the notation of the non-rigid correspondence noted on the diagram is helpful.

2.7. The reviewer is absolutely correct that morphVQ, like other learning-based approaches to morphological analyses, will introduce a range of nonlinear effects. While these discussions are important, we feel that this discussion will detract from the point of this paper - to demonstrate that a learning-based based on functional maps can adequately quantify surface morphology for biological analyses. As with GM and other approaches, the full statistical underpinnings have been explicated over many years (for example, see Felsenstein & others). We look forward to contributing to this discussion but feel strongly that it should be more deeply explored in separate manuscripts.

In the morphoVQ description, the operation of the Consistent ZoomOut process really needs more description of just what it means to "improve" point to point mappings.

2.8. All functional-map-based methods require a post-processing refinement step. In this paper, we apply methods of LimitShape (Huang 2019) and Consistent ZoomOut (Huang 2020) which builds on LimitShape for pair-wise shape matching for synchronizing elements across the shape collection. This method is very good for non-isometric biological shapes. In section 2.3 of the paper, we describe why we use ConsistentZoomOut, and in the methods we describe more details of Limit Shape. We refer readers to the appropriate literature for deeper understanding of this topic and its implementation.

The statement

“The Limit Shape method's CCLB matrices [40] provide canonically

consistent bases across different shape inputs, normalizing for shape-dependent

inconsistencies in the LBO.” (pg 15)

Again this seems to imply that is a non-linear operation? Is that correct? So this is second non-linear transformation (“shape dependent”??) being applied to the data at this point? If that is correct, I think the point needs to be emphasized that there are multiple non-linearities in the analysis chain.

It would help to have a carefully detail block diagram of the morphoVQ analysis chain included in the ms. Figure 1 is a bit too simple, and doesn't seem to show all the steps in sequence. Figure S1 is helpful in showing the operation of the feature extractor, but the Consistent ZoomOut procedure is really left as something of a black box, with presumably non-linear operation.

2.9. We have incorporated a new figure to explain the LimitShape and Consistent ZoomOut process.

Review Conclusions

This is good and important work. Some issues should be addressed prior to publication.

a.) The data set looks great, no concerns. A bit of discussion (or citation) of the impact of point number standardization on subsequent analyses would help.

b.) The analysis change morphoVQ is quite complex, and contains multiple non-linear transformations. I found it quite a demanding read to follow the process as presented here.

Some words of caution about the non-linears present and the impact of those on subsequent analyses seems wise.

c.) A number of the comparisons used in the paper compare data sets differing in the number of variables used (3DGM and Auto3D) with morphoVQ results decomposed into area and conformally components.

Some discussion of this issue, and why it was done, seems to be in order.

If at all possible, add a joint analysis of area and conformal analysis in the classification section.

2.10. We are grateful to this reviewer for recognizing the importance of this work and for their constructive critiques of our manuscript. We have incorporated their suggestions and they have clarified important aspects of the methods and their implementation.

Reviewer Three

The contribution by Thomas and colleagues is a highly welcomed one. Indeed, manual landmarking suffers from a long list of disadvantages, described in detail by the authors, while automated procedures are too few, and not devoid of problems of their own. Moving towards a landmark-less method of 3D shape analysis of digital model is indeed an extremely welcomed development. The authors manage to show that their FM framework-based method, incorporating shape descriptors learned using a deep neural network model, provides Genus-level classification that are equal to or better than traditional 3DGM methods.

What is missing in the article, in my opinion, is additional elaboration regarding some specific aspects of the method. I find that the entire discussion on the selected shape descriptors, as well as the methods through which they are obtained is not clear enough for myself (with working knowledge, albeit not expertise in common ML methods), and probably for a large portion of the readership. I would be more than happy to read more than two short paragraphs on the specificalities of spectral shape descriptors, wave kernel signature, SHOT (an abbreviation not explained in the text). I would also like to have more information in the Harmonic Surface Network used to extract the spectral descriptors. That means, not only its advantages over FMNet or SUREFMNet, or its specs provided in the implementation section, but a general description of how it works.

3. Thanks for providing this valuable comment. We have added a detailed description of HSN in how to generate shape descriptors in section 4.4. We also provide a short introduction and include citations for the SHOT and WKS in the last paragraph of section 4.3. However, we believe the in-depth description of SHOT and WKS is a bit out of scope of this paper given they are previous studies and in our paper we do not use them directly. So we decided not to include detailed descriptions for this two in this paper. Nevertheless, we make sure the necessary information can be achieved via citations in sections 4.3 and 4.4.

The decision not to use a validation set for testing the performance of the training for FMs, but simply relaying on convergence should be justified.

3.1. Since the current target is to show how well the model is able to generate new representations of shape variation in an automated fashion, and there is no ground truth correspondence information available for validation, we believe splitting the dataset set into training and testing set might not be helpful in demonstrating the performance of the model. Instead, we use different datasets to demonstrate that the model generalizes in different

situations. Additionally, we provide expert-labeled biological markers and compare the performance of the estimations to the biological ground truth landmarks. With these two maneuvers, we believe the performance of the model can be properly judged.
Nevertheless, we believe this comment is very important.

Auto3DGM is not the only automated landmark positioning procedure. A standalone software called AGMT3-D, which despite not being specifically designed for biological shape analysis, also allows to automatically position 3D pseudo-landmarks on triangulated mesh models.

3.2. Thank you for calling our attention to this reference. Since this paper is from the perspective of biology, we decide to keep the focus on the Auto3DGM. While we have looked into this paper this method will not be considered for official comparison.

The FMs provided by the model, despite being truncated, are still extremely high-dimensional. In my personal opinion it is OK to use them for exploring between-group differences, but maybe the authors should at least mention address the recent debate regarding the validity of results obtained in high p/n contexts (e.g. Bookstein 2019, Goswami et al. 2019, Cardini 2020, Rholf 2021).

3.3. This is an important issue that warrants further discussion and investigation. However, there is little consensus among practitioners within the GM framework regarding how to resolve or address the problem of [underdetermination]. Exploring this topic properly requires more space than we can allot in this manuscript and we intend to focus on it in a future analysis that can more thoroughly address strategies for dealing with this with LSSDs and function-based methods.
Despite having a high p/n in a highly complex model, we provide a validation experiment in our revised manuscript to compare the landmark estimations with the expert labeled ground-truth to demonstrate our model can infer biologically (See validation experiment results in the newly added section 2.8 and also section 4.9 in the method section).

One cannot assert that a new method is more computationally efficient than an old one without showing any data on runtime or computational costs of training/running the model.

3.4. An Auto3DGM analysis of the same shape collection with 512 pseudolandmarks took 26 days to complete, our pipeline took 2 days.

I could not observe figures 6, 7 – because "The image file is corrupt or invalid".

3.5. This has been fixed in the revision.

Reviewer Four

This paper introduces a new pipeline for morphological analysis for 3D shapes in a fully automatic way using the functional map framework. Rather than relying on manually specified landmarks, the authors propose to use a fully automatic correspondence estimation pipeline that was developed in the geometry processing/vision communities and demonstrate its use in geometric morphometrics. The main novelty of this paper, therefore, is to demonstrate the applicability of this automatic pipeline for both phenotyping and for highlighting areas of variability. The main application is for analyzing hominoid cuboids, and using both the automatically detected correspondences and the LSSDs (which capture shape variability in a collection of shapes linked by functional maps) for fully automatic morphological phenotyping.

I am not an expert in geometric morphometrics (my expertise is mainly in 3D shape analysis), but I thought both the motivation and the technical execution of the paper were convincing. The authors have done their due diligence in investigating and adapting the latest relevant developments in the functional map literature to their own problem setting and have demonstrated a compelling evaluation compared to baseline approaches, both manual and automatic. I particularly appreciated the use of unsupervised learning for computing features by adapting the SURFMNet architecture to the given shape collection, while using the HSN feature extractor. Furthermore, the authors used the very recent consistent zoomout and Latent Space Shape Differences (LSSDs) to encode shape variability. These are non-trivial concepts that are all nicely brought together in this work.

4. Thanks for the appreciation of this work. We believe this proposed method could provide additional insights and useful cases in the shape analysis in biology.

I have a few relatively minor comments and suggestions:
1. The authors mention that their shapes should be approximately rigidly aligned ("Our algorithm performs best when polygon models are approximately rigidly aligned..."). However, later on the authors also mention that they adopt the HSN feature extractor because it is capable of producing rotationally-invariant features. "This is usually the case for real-world pairs of bone meshes which may be poorly aligned, if at all." These two statements seem contradictory -- if the shapes need to be pre-aligned anyway, what is the advantage of using HSN? Furthermore, in some functional map learning frameworks such

as [60], data augmentation is used to achieve rotation invariance. I think it's important to comment on exactly how much rotation invariance is needed and where it plays most role.

4.1. Thanks for pointing out this. The "rigidly aligned" term and the "rotationally-invariant" term are not contradictory in this case. The HSN feature extractor has a rotation invariant and the rotation equivariant kernel. A structure that has these two types of kernels does possess full rotation invariance due to the equivariant part. However, this HSN structure is still able to have some level of rotation invariance (e.g. +- 5 degrees in our experiments). This is why we still pre-aligned the shapes. In summary, due to the introduction of the rotation invariant kernel in HSN, this model tolerates a wider range of shapes when they are not aligned perfectly. To make this clarification, we have added content in the new section 4.4 for describing the HSN in details and also reported the number of rotation degrees in section 4.5.1.

The functional map pipeline has recently been used for morphometric analysis in "Geometric analysis of shape variability of lower jaws of prehistoric humans" by Ren et al. That work should be cited and discussed at least in some detail, as it seems to tackle similar goals as presented in this submission. Furthermore, even the code of that work is publicly available at https://github.com/llorz/PROJ2020_jaws Therefore, it would be reasonable to expect a comparison to that work as another baseline. It would be interesting and useful to know which sets of approaches are most accurate and robust.

4.2. Thanks a lot for pointing out this reference. In our paper, we included the ZoomOut paper, Consistent ZoomOut paper and the Limit Shape paper. All the three papers come from the same group as this reference pointed out here. And we believe the three papers we cited are published after this work, thus should be enough to cover this perspective in terms of the geometric shape matching. Additionally, we include the description of the Consistent Zoomout and the LimitShape in our revised manuscript this time to give more details on these techniques (see section 4.6).
In terms of the additional baseline issue, since this paper is focused on the power of our proposed MorphVQ versus the conventional tool used in biological shape analysis (e.g. Auto3DGM), there are more experiments and discussions needed the evaluation of the level of biological interpretability with this model inference, and hence out of scope of this paper. As a result, we did not include additional baselines solely for geometric shape matching. Another reason that let us not add additional baseline, as pointed out in this reference, is that the Consistent ZoomOut, which is believed as the successor of this reference, has covered the corresponding discussion already (refer to section 4.6).

Some citations seem to be not properly formatted. For example, [62] is missing the venue. The same is true for many other references, such as [58], [21] and others. Furthermore, some titles are wrong such as "A Functional View of Geometry Processing" should both include a venue and should not have an article at the beginning. A careful proof-reading of the bibliography and fixing all the references would be useful.

4.3. Thanks for the advice, this is fixed now in the revision.

The authors rely on the Latent Space Shape Differences, which seem to arise from a paper that is only on arxiv. Instead another paper "Limit shapes–a tool for understanding shape differences and variability in 3d model collections" published by the same authors has appeared in the Computer Graphics Forum. In that paper the authors define Characteristic Shape Differences, which correspond to the same concept. If there is any distinction between these concepts, it would be useful to state it explicitly. If not, I would suggest the authors to use the terminology from the more recent, published work.

4.4. Thanks for the advice. Two papers have no difference in terms of the scope and coverage of the technique as discussed in this paper. As a result, we updated the citations to include published work in the revision. (cite new limit shape paper number)

Despite these minor concerns, I think this paper makes a valuable contribution, both by adapting the functional map framework to a novel domain and by introducing a fully automatic morphological phenotyping technique. I therefore believe that this paper is likely to inspire future work.

Finally, I have not checked but it would be very useful if the authors released their data and code in order to facilitate further research in this area.

4.5. We are grateful to this reviewer for their constructive feedback and appreciation of the novel contributions that our work makes to the field of morphological analysis.
