# Supplementary material for: Automated morphological phenotyping using learned shape descriptors and functional maps: A novel approach to geometric morphometrics
Source: PLoS Comput Biol. 2023 Jan 19;19(1):e1009061. doi: 10.1371/journal.pcbi.1009061 (PMC9970057; doi:10.1371/journal.pcbi.1009061)
Supplement: Attachment — Submitted filename: PCOMPBIOL-D-21-00802_R1_Review_Notes.pdf [file pcbi.1009061.s008.pdf]

From the response to Reviewer Two:

Original Reviewers Comments

The morphoVQ method (see discussion below) was used to extract two sets, one conformal (angular based) and one area-based LSSDs (LSSD- Latent Shape Space Difference). The differences in shapes are thus decomposed by the Functional map network (FMN) approach into measures that represent differences in areas of the surfaces and differences in relative orientation or angle. It appears that two morphoVQ data sets are used, one representing conformal changes and one representing area differences From my perspectives the use of this decomposition of the shape differences in morphoVQ into the conformal (angular) and area-based result needs more discussion.

The authors compare the 3DGM and Auto3D methods at different data point densities (numbers of points) to the morphoVQ data sets which differ not in the number of points used, but in the nature or decomposition of the shape differences. In the 3DGM and Auto3D approaches, the two set sets in each case differ in the number of points, but in the morphoVQ, the sets used differ not in the number of points or variables used, but rather in the nature of shape differences (conformal or areal) employed.

The sub-setting of the 3 data types is not consistent across the 3 analysis methods. Why are the conformal and area results in morphoVQ split into two discrete data sets and then analyzed separately? They represent different types of shape differences, why are they treated separately?

Surely it is possible to combine the area and conformal data about shape changes into a unified analysis when carrying out the classification task? The ability to split shape differences into the different types of alteration (conformal and area) may be of some real use (as decompositions often are), but it is not clear why data set differing in decomposition (the morphoVQ) should be compared to data differing in point density (3DGM and Auto3D).

Please explain in detail why this is done, and/or show comparisons among data sets that differ simply in point density.

If morphVQ cannot be used without the decomposition step, or if the two decomposition products cannot or should not be treated separately. 2.2.

The morphVQ method generates two different representations, conformal (reflecting angular changes on surface) and area-based (quantifying relative-sizes of portions of the bone). These differences are described in the introduction. These two methods allow identification of where and why bone shapes differ, thus we present figures to illustrate the cuboid regions where conformal and area-based regions differ between genera. See figures 8 and 9. This means of representing shape differences as resulting from differential distortions of area or angularity provides a new method to address a longstanding problem in comparative morphology.

Reviewer Response to Author's comments

As stated in the introduction of the paper, the two different representations of shape produced by this method, the conformal and area-based methods are indeed providing a new method to address a long-standing problem, a potentially valuable approach.

However, the authors are comparing this new approach of splitting these two aspects of shape against methods that do not carry out this type of decomposition of the shape information into two distinct components, specifically 3DGM and Auto3D.

This is not a comparison of like variable sets to one another, the MorphoVQ introduces a novel decomposition of shape into two terms not used elsewhere.

The authors have declined to make a simple and modest alteration (a comparison of the two aspects combined of the MorphoVQ method to the other methods). This leaves the reader unable to determine what impact if any this additional decompositional aspect of MorphoVQ might be having on the results.

Approaches to adding this comparison are very simple relative to the work completed in the paper, I don't understand the author's reluctance on this issue. When you present a fundamentally new method, complete comparisons to previous practice greatly improve the utility of the paper and the new method.

This is a complex paper that places a lot of demands on the reader, clean the loose ends to maximize the value to the reader.

The paper was substantially approved in the revised version, but this issue remains to be addressed.
