# Supplementary material for: Automated morphological phenotyping using learned shape descriptors and functional maps: A novel approach to geometric morphometrics
Source: PLoS Comput Biol. 2023 Jan 19;19(1):e1009061. doi: 10.1371/journal.pcbi.1009061 (PMC9970057; doi:10.1371/journal.pcbi.1009061)
Supplement: Attachment — Submitted filename: Response to Reviewers.pdf [file pcbi.1009061.s009.pdf]

ILLINOIS

COLLEGE OF LIBERAL ARTS & SCIENCES

Department of Anthropology
109 Davenport Hall, MC-148
607 S. Matthews Ave.
Urbana, IL 61801-3636

July 20th, 2022

Dear Editors Xu and Ma,

On behalf of all of the authors, we would like to thank you and the reviewers once more for your thorough review of our manuscript. We have carefully considered and addressed the new issues suggested by the reviewers and have detailed our responses below. As requested, we have attached a marked-up version of the original manuscript and a clean version incorporating all changes.

We trust that our changes and comments in the responses below have addressed all of the reviewers' concerns.

Sincerely,

Oshane O. Thomas
Ph.D. Candidate | Department of Anthropology
University of Illinois at Urbana-Champaign

Revisions

Reviewer One

The authors have made a commendable effort in replying to prior criticism. In general, I do find the approach ingenious and its comparison to Auto3DGM appropriate. I think, however, that some of my prior concerns have not been truly addressed.

In particular, I still think this manuscript relies on topologically simple skeletal structures, in which homology is hard to define. Another way of putting this would be: how will this method fare with a structure such as the mammalian skull, where sutures delineate sharp homology boundaries? My intuition is that the current approach would not reflect any notion of biological homology, despite reflecting general geometric similarity.

1. We agree with the reviewer to some extent as our method does work best when differences in a morphological collection are dominated by distinctive differences in surface curvature among specimens. If a collection of mammalian skulls were considered, we agree that features delineated by shape homology boundaries would be difficult to capture. We want to note however that Auto3DGM and other automated point-based methods that do not depend on manually identified points would also suffer as well.

That is the reason why I emphasized landmark locations in the previous iteration. I do not think landmarks themselves are a better approach for describing shape variation. I simply think that the manuscript needs to clearly evaluate the extent to which these geometric correspondences reflect any notion of biological homology. I still cannot answer that question, given the current version of the manuscript.

1.1. The validation study presented in our last revision shows that we can capture landmark-based homology with greater accuracy than Auto3DGM as we are able to recover type-I and type-II landmark locations remarkably well. While we have not yet performed such an experiment with outline-based quantification, we expect our approach to perform equally well, especially on larger collections of shapes.

Having said that, I do understand that I am mostly alone in my concerns, with other reviewer's being mostly positive about the manuscript. I sincerely hope, however, that the authors will take these concerns seriously.

1.2. The issues you've raised and your concern regarding biological homology are ones we have considered extensively. In fact, they have been driving further research for some time. However, this paper introduces one of the first deep-learning- and functional-map-based approaches to characterizing biological shape correspondence. While the comparisons and experiments are conducted across different collections of shapes, our fully automated approach might not be suitable for all morphological datasets, especially those that are more topologically complex.

We are currently working on a semi-automated approach more suited to topologically complex forms that use manually coded features captured on a small subset of a shape collection. This semi-supervised learning-based approach is a whole other question and is worthy of its own manuscript. While the fully automated approach we present here works well for all skeletal elements except the cranium, we don't consider it the only functional-map-based approach to solving this complex problem.

Geometry processing has advanced considerably since the submission of this manuscript. We believe this growing toolkit will continue to surprise us with new ways to quantify, represent, and explore shape variation in the future.

Reviewer Two

I went over the revised version of the manuscript and the authors rebuttable to my previous comments. Indeed, it appears that all of my most major concerns have been adequately addressed, while a sufficient explanation was provided for those which were not. Therefore, I recommend the paper to be published in POLS computational biology.

2. Thank you once again for your thorough review and for your final recommendation.

Reviewer Three

As stated in the introduction of the paper, the two different representations of shape produced by this method, the conformal and area-based methods are indeed providing a new method to address a long-standing problem, a potentially valuable approach.

However, the authors are comparing this new approach of splitting these two aspects of shape against methods that do not carry out this type of decomposition of the shape information into two distinct components, specifically 3DGM and Auto3D. This is not a comparison of like variable sets to one another, the MorphoVQ introduces a novel decomposition of shape into two terms not used elsewhere.

The authors have declined to make a simple and modest alteration (a comparison of the two aspects combined of the MorphoVQ method to the other methods). This leaves the reader unable to determine what impact if any this additional decompositional aspect of MorphoVQ might be having on the results. Approaches to adding this comparison are very simple relative to the work completed in the paper, I don't understand the author's reluctance on this issue. When you present a fundamentally new method, complete comparisons to previous practice greatly improve the utility of the paper and the new method. This is a complex paper that places a lot of demands on the reader, clean the loose ends to maximize the value to the reader.
The paper was substantially approved in the revised version, but this issue remains to be addressed.

3. We apologize for excluding the requested analysis from our last revision. This was done in error. We have updated Table 1 to include the classification results of combined Area-based and Conformal LSSDs features. We also note in our results section 2.6 that this combined analysis yields classification accuracies similar to those obtained for each representation.

Reviewer Four

This is a revision of the paper that I reviewed previously. The authors have incorporated the majority of all of the required changes and have substantially revised the manuscript. The cover letter and summary of changes were particularly useful. The main changes with respect to the previous version include demonstrating the performance of the proposed approach on two other datasets analyzing shape variation in mouse humeri and in primate medial cuneiforms. This substantially improves the scope and impact of the proposed method and highlights its general power.

Overall, I find this paper of significant value as it aims to address a fundamental problem in Geometric Morphometrics, reducing the need for manual intervention and helping to avoid annotation bias. As such, I think it can open the door to future works and constitutes an important contribution to the field.

When preparing the final version, I only have two suggestions for the authors:

*1. I would like to request a more detailed discussion about the importance of rotation invariance in the analysis process. Specifically, I did not quite understand the response provided by the authors in the cover letter about this question. "Rotation-invariance" is a technical term that means that the feature extractor should produce the same exact features regardless of the orientation in space. It seems that the authors advocate the use HSN because it is *robust* with respect to small orientation changes. The authors mention "However, this HSN structure is still able to have some level of rotation invariance (e.g. +- 5 degrees in our experiments)." This to me*

is not quite clear, since a rotationally-invariant feature extractor should produce exactly the same features regardless of orientation. The sentence by the authors, rather, suggests "robustness" with respect to small orientation changes, instead of true rotational "invariance." I think this should be clarified.

4. We thank the reviewer for pointing out this ambiguity. We included a description on the rotation invariance and equivariance, and rephrased some of the sentences in Section 4.5.1.

2. Since the authors emphasize the efficiency of their approach, it would be worthwhile to add timings for different stages of their approach. Perhaps I missed it, but I could not a detailed timing report. In the cover letter the authors mention "An Auto3DGM analysis of the same shape collection with 512 pseudolandmarks took 26 days to complete, our pipeline took 2 days. " This definitely has to be reported in the manuscript and also I would suggest to report the processing cost of different stages in the algorithm to see what parts take the most amount of time. This could help to appreciate the efficacy and bottlenecks of the proposed approach.

4.1. We thank the reviewer for pointing out this missing piece. We have added results section 2.9 to briefly describe the difference in speed between our approach and Auto3DGM.

Apart from these minor comments, I think the manuscript is in great shape and I would be happy to recommend its acceptance.
