# Supplementary material for: Automated morphological phenotyping using learned shape descriptors and functional maps: A novel approach to geometric morphometrics
Source: PLoS Comput Biol. 2023 Jan 19;19(1):e1009061. doi: 10.1371/journal.pcbi.1009061 (PMC9970057; doi:10.1371/journal.pcbi.1009061)
Supplement: Attachment — Submitted filename: Response to Reviewers - Updated Data Availability Statement.pdf [file pcbi.1009061.s010.pdf]

ILLINOIS

COLLEGE OF LIBERAL ARTS & SCIENCES

Department of Anthropology
109 Davenport Hall, MC-148
607 S. Matthews Ave.
Urbana, IL 61801-3636

November 5th, 2022

Dear Editors Xu and Ma,

On behalf of all of the authors, we would like to thank you and the reviewers once more for your thorough review of our manuscript and we are delighted to hear that you are likely to publish our work.

I would like to apologize for not responding to you in a timely manner, I will strive to do just that from now on.

The current manuscript being submitted now includes mention of the dryad repository in the methods section and the dryad citation added to the references section. Please let me know if you need any additional information or if I need to make any other changes.

Thank you again for your time and consideration.

Sincerely,

Oshane O. Thomas
Ph.D. Candidate | Department of Anthropology
University of Illinois at Urbana-Champaign
