# Supplementary material for: Automated morphological phenotyping using learned shape descriptors and functional maps: A novel approach to geometric morphometrics
Source: PLoS Comput Biol. 2023 Jan 19;19(1):e1009061. doi: 10.1371/journal.pcbi.1009061 (PMC9970057; doi:10.1371/journal.pcbi.1009061)
Supplement: Attachment — Submitted filename: Review_PComBio.docx [file pcbi.1009061.s005.docx]

The contribution by Thomas and colleagues is a highly welcomed one. Indeed, manual landmarking suffers from a long list of disadvantages, described in detail by the authors, while automated procedures are too few, and not devoid of problems of their own. Moving towards a landmark-less method of 3D shape analysis of digital model is indeed an extremely welcomed development. The authors manage to show that their FM framework-based method, incorporating shape descriptors learned using a deep neural network model, provides Genus-level classification that are equal to or better than traditional 3DGM methods.

What is missing in the article, in my opinion, is additional elaboration regarding some specific aspects of the method. I find that the entire discussion on the selected shape descriptors, as well as the methods through which they are obtained is not clear enough for myself (with working knowledge, albeit not expertise in common ML methods), and probably for a large portion of the readership. I would be more than happy to read more than two short paragraphs on the specificalities of spectral shape descriptors, wave kernel signature, SHOT (an abbreviation not explained in the text). I would also like to have more information in the Harmonic Surface Network used to extract the spectral descriptors. That means, not only its advantages over FMNet or SUREFMNet, or its specs provided in the implementation section, but a general description of how it works.

Some more specific points:

- The decision not to use a validation set for testing the performance of the training for FMs, but simply relaying on convergence should be justified.
- Auto3DGM is not the only automated landmark positioning procedure. A standalone software called AGMT3-D, which despite not being specifically designed for biological shape analysis, also allows to automatically position 3D pseudo-landmarks on triangulated mesh models.
- The FMs provided by the model, despite being truncated, are still extremely high-dimensional. In my personal opinion it is OK to use them for exploring between-group differences, but maybe the authors should at least mention address the recent debate regarding the validity of results obtained in high p/n contexts (e.g. Bookstein 2019, Goswami et al. 2019, Cardini 2020, Rholf 2021).
- One cannot assert that a new method is more computationally efficient than an old one without showing any data on runtime or computational costs of training/running the model.
- I could not observe figures 6, 7 – because “The image file is corrupt or invalid”.

All in all, I think this is a very good manuscript and hence I recommend it to be accepted for publication, following the authors addressing the minor comments provided above.

References cited:

Cardini, A., 2020. Less tautology, more biology? A comment on “high-density” morphometrics. *Zoomorphology*, *139*(4), pp.513-529.

Goswami, A., Watanabe, A., Felice, R.N., Bardua, C., Fabre, A.C. and Polly, P.D., 2019. High-density morphometric analysis of shape and integration: the good, the bad, and the not-really-a-problem. *Integrative and Comparative Biology*, *59*(3), pp.669-683.

Bookstein, F.L., 2019. Pathologies of between-groups principal components analysis in geometric morphometrics. *Evolutionary Biology*, *46*(4), pp.271-302.

Rohlf, F.J., 2021. Why Clusters and Other Patterns Can Seem to be Found in Analyses of High-Dimensional Data. *Evolutionary Biology*, *48*(1), pp.1-16.
