# Supplementary material for: Automated morphological phenotyping using learned shape descriptors and functional maps: A novel approach to geometric morphometrics
Source: PLoS Comput Biol. 2023 Jan 19;19(1):e1009061. doi: 10.1371/journal.pcbi.1009061 (PMC9970057; doi:10.1371/journal.pcbi.1009061)
Supplement: Attachment — Submitted filename: Review_PCOMPBIOL-D-00802.docx [file pcbi.1009061.s006.docx]

Review of

Automated morphological phenotyping using learned shape

descriptors and functional maps: A novel approach to

geometric morphometrics

Oshane O. Thomas, Hongyu Shen, Ryan L. Raaum William E.H.

Harcourt-Smith John D. Polk, Mark Hasegawa-Johnson

**General Overview**

This paper presents a pipeline system, the Morphological Variation Quantifier (morphoVQ) an analysis pipeline (or collection of processing steps) that is meant to automate the extraction of variables from 3D scans of objects, typically structures of biological objects, hominoid cuboid bones in this case.

The current common approach to extracting variables from these types of 3D scans is 3D landmark based geometric morphometrics (3DGM), which requires a skilled and knowledgeable human operator to digitize landmark and/or semi-landmark locations on each specimen, which is a costly process. There is also at least one currently existing automated approach to extracting variables for comparison, Auto3DGM.

The paper compares the performance of the newly developed analysis pipeline morphoVQ to the results obtained using the other two methods. While it is difficult in this situation to have a “ground truth”, the human operator based 3DGM (with or without the use of semi-landmarks) at least represents something of a standard or accepted state of the art approach (albeit labor intensive).

The paper looks at the classification of specimens using K-means, logistic regression, a Naïve Bayes classifier and a Linear Discriminant Analysis on PCA axes scores derived from the 6 distinct data sets. Visual representations of where the variance is most pronounced are also presented.

**Data, and Data Subsets**

The data was 102 laser surface scans of hominoid cuboid bones, which had vertex counts from 2000-39,000 points, these were interpolated or reduced to a standardized 12,000 vertices using a common algorithm. One hopes that the possible impact of this standardization of vertex count has been studied previously, and/or looked at some detail with regards to it’s impact on subsequent analyses.

Both 3DGM and Auto3DGM were carried out on this data, producing two variations on each method. In 3DGM one approach bused 21 LMs, while the second added SLMs for a total of 130 points. AutoGM was set to create two sets, 256 and 512 pseudo-landmarks.

The morphoVQ method (see discussion below) was used to extract two sets, one conformal (angular based) and one area based LSSDs (LSSD- Latent Shape Space Difference). The differences in shapes are thus decomposed by the Functional map network (FMN) approach into measures that represent differences in areas of the surfaces and differences in relative orientation or angle. It appears that two morphoVQ data sets are used, one representing conformal changes and one representing area differences

From my perspectives the use of this decomposition of the shape differences in morphoVQ into the conformal (angular) and area-based result needs more discussion. The authors compare the 3DGM and Auto3D methods at different data point densities (numbers of points) to the morphoVQ data sets which differ not in the number of points used, but in the nature or decomposition of the shape differences.

In the 3DGM and Auto3D approaches, the two set sets in each case differ in the number of points, but in the morphoVQ, the sets used differ not in the number of points or variables used, but rather in the nature of shape differences (conformal or areal) employed. The sub-setting of the 3 data types is not consistent across the 3 analysis methods.

Why are the conformal and area results in morphoVQ split into two discrete data sets and then analyzed separately? They represent different types of shape differences, why are they treated separately? Surely it is possible to combine the area and conformal data about shape changes into a unified analysis when carrying out the classification task?

The ability to split shape differences into the different types of alteration (conformal and area) may be of some real use (as decompositions often are), but it is not clear why data set differing in decomposition (the morphoVQ) should be compared to data differing in point density (3DGM and Auto3D). Please explain in detail why this is done, and/or show comparisons among data sets that differ simply in point density.

If morphVQ cannot be used without the decomposition step, or if the two decomposition products cannot or should not be treated separately.

**Shape Space Comparison**

The shape spaces are compared using Pearon’s r.

What variables are used in the computation of Pearson’s r? Is this a multivariate form of R, using one set of shape variables to predict another? Or is it the r based on estimated intra-specimen distances? I think this section needs to be a bit more explicit on exactly how this was done.

It was rather interesting that the range of r values reported was very narrow, ranging from 0.639 to 0.616. This narrow strikes me as a bit odd, in that all the variations in data produce such a narrow range of correlations around a moderate level of correlation. Is there any way to see what correlation arises from a randomization process on this data?

If all the correlations were uniformly high (up over 0.9), I would understand the compressed range, but the grouping of all the correlations around the same value seems odd. It does appear that the area and conformal results (split by the decomposition) are being compared to the non-decomposed 3DGM and Auto3D results, is that correct?

**Classification Results**

This looks to be well done, I’m glad to see a fold-based cross validation used here.

Again, I’d like to see an analysis of the combined Area and conformal data sets here, it might show even higher performance.

If you revise this section, I’d suggest trying a quick random forest classifier and/or an SVM classifier, they seem to outperform the earlier methods, at least with some data sets. Once you have the data reduced to PCA scores, running a couple of other classifier methods is a pretty trivial operation and may give you some higher rates.

**Figures**

In figures 6 and 7, I assume the columns in these figures have the same labels as they do in figure 5? Please add the column labels to the figures, it just makes them easier to interpret.

Figures 6 and 7 are showing Area-based and conformal based differences using the 3 different methods. How were the area and conformal information extracted from the 3DGM and Auto3D methods? Did I miss this in the ms? Please explain this in detail, or simply let me know if I missed this.

**The morphoVQ method**

The morphoVQ method is a pipeline of series of operations to extract Latent Shape Space Differences (LSSDs) through a lengthy set of operations. The Deep HSN UResNet Feature extractor process used as a feature extractor is using some non-linear elements, so this process is fairly non-linear.

The now-standard 3DGM also has a non-linear operation, the division by a centroid size measure. A lot of effort in the development of GM methods focused on the nature and impact of non-linear operations on the distribution of variance in these systems. When scaling by size, the consistent use of centroid size at least meant a consistent non-linear effect on the data, uniform across studies. That will not be the case with the morphoVQ method.

Early work on 3DGM was in the context of a statistical world where resampling/permutation and cross validation were just starting to see substantial use. Use of GM data with traditional statistical methods with substantial distributional assumptions embedded within them meant that non-linearity in data processing (such as division by centroid size or other scaling factors) produced alterations in the underlying distributions of variance that posed substantial concerns, particularly when using traditional statistical approaches with distributional assumptions.

I think some discussion of the impact of the extensively non-linear nature of this method is warranted, perhaps there is some available literature in this area that could be cited. This is particularly critical for applied workers. Figure 1 does help, the notation of the non-rigid correspondence noted on the diagram is helpful.

In the morphoVQ description, the operation of the Consistent ZoomOut process really needs more description of just what it means to “improve” point to point mappings.

The statement

“The Limit Shape method's CCLB matrices [40] provide canonically

consistent bases across different shape inputs, normalizing for shape-dependent

inconsistencies in the LBO.” (pg 15)

Again this seems to imply that is a non-linear operation? Is that correct? So this is second non-linear transformation ( “shape dependent”??) being applied to the data at this point? If that is correct, I think the point needs to be emphasized that there are multiple non-linearities in the analysis chain.

It would help to have a carefully detail block diagram of the morphoVQ analysis chain included in the ms. Figure 1 is a bit too simple, and doesn’t seem to show all the steps in sequence. Figure S1 is helpful in showing the operation of the feature extractor, but the Consistent ZoomOut procedure is really left as something of a black box, with presumably non-linear operation.

**Review Conclusions**

This is good and important work. Some issues should be addressed prior to publication.

a.) The data set looks great, no concerns. A bit of discussion (or citation) of the impact of point number standardization on subsequent analyses would help.

b.) The analysis change morphoVQ is quite complex, and contains multiple non-linear transformations. I found it quite a demanding read to follow the process as presented here.

Some words of caution about the non-linears present and the impact of those on subsequent analyses seems wise.

c.) A number of the comparisons used in the paper compare data sets differing in the number of variables used (3DGM and Auto3D) with morphoVQ results decomposed into area and conformally components.

Some discussion of this issue, and why it was done, seems to be in order.

If at all possible, add a joint analysis of area and conformal analysis in the classification section.
